# Transformation of sugarcane molasses into fructooligosaccharides with enhanced prebiotic activity using whole-cell biocatalysts from *Aureobasidium pullulans* FRR 5284 and an invertase-deficient *Saccharomyces cerevisiae* 1403-7A

**DOI:** 10.1186/s40643-021-00438-7

**Published:** 2021-09-03

**Authors:** Most Sheauly Khatun, Morteza Hassanpour, Solange I. Mussatto, Mark D. Harrison, Robert E. Speight, Ian M. O’Hara, Zhanying Zhang

**Affiliations:** 1 Centre for Agriculture and the Bioeconomy, Faculty of Science, Queensland University of Technology, Brisbane, QLD 4000 Australia; 2 School of Mechanical, Medical and Process Engineering, Faculty of Engineering, Queensland University of Technology, Brisbane, QLD 4000 Australia; 3 Department of Biotechnology and Biomedicine, Technical University of Denmark, Søltofts Plads, Building 223, 2800 Kongens Lyngby, Denmark; 4 School of Biology and Environmental Science, Faculty of Science, Queensland University of Technology, Brisbane, QLD 4000 Australia; 5 ARC Centre of Excellence in Synthetic Biology, QUT, Brisbane, QLD 4000 Australia

**Keywords:** Sugarcane molasses, *A. pullulans*, Transfructosylating activity, Fructooligosaccharides, Prebiotics, Probiotics

## Abstract

Fructooligosaccharides (FOS) can be used as feed prebiotics, but are limited by high production costs. In this study, low-cost sugarcane molasses was used to produce whole-cell biocatalysts containing transfructosylating enzymes by *Aureobasidium pullulans* FRR 5284, followed by FOS production from molasses using the whole-cells of *A. pullulans*. *A. pullulans* in molasses-based medium produced cells and broth with a total transfructosylating activity of 123.6 U/mL compared to 61.0 and 85.8 U/mL in synthetic molasses-based and sucrose-based media, respectively. It was found that inclusion of glucose in sucrose medium reduced both transfructosylating and hydrolytic activities of the produced cells and broth. With the use of pure glucose medium, cells and broth had very low levels of transfructosylating activities and hydrolytic activities were not detected. These results indicated that *A. pullulans* FRR 5284 produced both constitutive and inducible enzymes in sucrose-rich media, such as molasses while it only produced constitutive enzymes in the glucose media. Furthermore, treatment of FOS solutions generated from sucrose-rich solutions using an invertase-deficient *Saccharomyces* yeast converted glucose to ethanol and acetic acid and improved FOS content in total sugars by 20–30%. Treated FOS derived from molasses improved the in vitro growth of nine probiotic strains by 9–63% compared to a commercial FOS in 12 h incubation. This study demonstrated the potential of using molasses to produce FOS for feed application.

**Supplementary Information:**

The online version contains supplementary material available at 10.1186/s40643-021-00438-7.

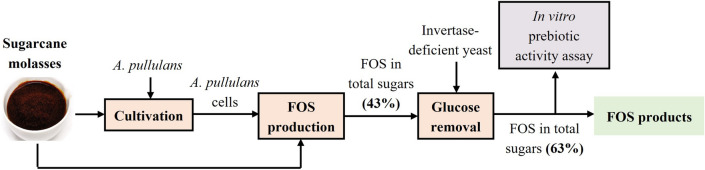

## Introduction

Fructooligosaccharides (FOS) are used as prebiotics in food and feed (Bali et al. 2015; Flores-Maltos et al. 2016). FOS are produced by transfructosylating enzymes, *β*-D-fructofuranosidase (FFase, EC 3.2.1.26) and fructosyltransferase (FTase, EC 2.4.1.9). FFase is a type of invertase, and it mainly hydrolyzes sucrose into glucose and fructose. Some FFases exhibit significant transfructosylating activities and produce FOS from sucrose through two reaction pathways: reverse hydrolysis and transfructosylating (Antosova and Polakovic 2002). The FOS concentration depends on the rates of synthesis and hydrolysis reactions. In general, FFases having high ratios of transfructosylating activity (U_t_) to hydrolytic activity (U_h_) are preferred for producing FOS with relatively high yields (Yoshikawa et al. 2007). In contrast, FTase mainly has U_t_ and its hydrolytic activity is very low because of its low affinity towards water as an acceptor (Antosova and Polakovic 2002).

Filamentous fungi such as *Aspergillus* strains and yeast such as *Aureobasidium* strains are the most studied microorganisms to produce transfructosylating enzymes for FOS production (Bali et al. 2015; Flores-Maltos et al. 2016). Transfructosylating enzymes can be constitutive enzymes which are always produced with the growth of the microorganisms, or inducive enzymes which are only expressed in the presence of inducers. A wide range of carbon sources, such as sucrose, glucose and glycerol have been used to produce transfructosylating enzymes by filamentous fungi and yeast (Bali et al. 2015; Flores-Maltos et al. 2016). Sucrose is the most commonly used carbon source for producing transfructosylating enzymes. For constitutive transfructosylating enzymes, sucrose is often an inducer, which not only induces U_t_, but also U_h_. Furthermore, microorganisms produce mixed transfructosylating enzymes, which may include both constitutive and inducible enzymes. For example, *A. pullulans* DSM 2404 (ATCC 9348) produced five types of FFases, one of which was constitutively expressed, and its expression was not inhibited by glucose (Yoshikawa et al. 2006; 2007). In another study, one *Aspergillus* strain cultivated on glucose-based medium led to the highest U_t_ among several carbon sources including glucose, sucrose and raffinose (Nascimento et al. 2019).

Molasses is a liquid by-product from sucrose manufacture in sugar mills and contains 30–60% sucrose, around 6–12% glucose, and 6–12% fructose (Bortolussi and O’Neill 2006; Dorta et al. 2006; Zhang et al. 2019). Since many microorganisms produce both constitutive and inducible transfructosylating enzymes, it is expected that a carbon source containing mixed sugars such as molasses will affect enzyme activity differently from the use of individual sugars. While molasses has been previously used to produce transfructosylating enzymes by *Aspergillus* strains (Dorta et al. 2006; Xie et al. 2017), the effect of mixed sugars in molasses on producing transfructosylating enzyme has not been studied.

Conversion of pure sucrose to FOS by transfructosylating enzymes has been well-studied and several studies also reported the use of molasses for synthesis of FOS (Khatun et al. 2020; Shin et al. 2004; Zhang et al. 2019). Transformation of low-value molasses to high-value prebiotic FOS as feed additives will enhance the revenue to the sugar industry. Furthermore, FOS-derived from molasses has the potential to be used directly as a feed additive as molasses itself is already a feed supplement. However, the presence of FOS production by-products, especially glucose, could reduce the FOS content and delivery efficiency. In order to improve FOS purity, produced FOS are often purified using relatively expensive separation and purification processes such as ultra-filtration and chromatographic separation (Crittenden and Playne 2002; Nobre et al. 2014, 2012; Pinelo et al. 2009). However, for feed application, the use of these expensive purification methods may not be necessary. Alternatively, microbial treatment, which selectively removes glucose (and fructose) without hydrolyzing FOS, may be a more practical and low-cost approach to prepare feed-grade FOS. Although there have been studies on the production of FOS from molasses (Shin et al. 2004; Zhang et al. 2019), the use of microbial treatment to improve molasses FOS content and the assessment of prebiotic activity of molasses-derived FOS have not been previously reported.

In the present study, the effect of glucose in sugarcane molasses on U_t_ and U_h_ of transfructosylating enzymes produced by a novel *A. pullulans* strain was evaluated. Furthermore, whole-cell biocatalysts of *A. pullulans* were then used to transform molasses into FOS. A simple microbial treatment step by using invertase-deficient yeast cells was then applied to the resulting FOS-containing to remove reducing sugars. Following microbial treatment, the treated FOS solutions were evaluated for in vitro prebiotic activity using nine potential probiotic strains without further purification. The prebiotic activities of molasses-derived FOS were compared with those of high-purity commercial FOS and FOS produced from pure sucrose. In addition, non-FOS sugar solutions (glucose, sucrose, molasses, and synthetic molasses) were also included in the prebiotic assay to assess the preferences of these probiotics on different sugar sources.

## Materials and methods

### Materials

Sugarcane molasses was collected from the Racecourse Sugar Mill in Mackay, Australia. Molasses contained 41.7 wt% sucrose, 7.4 wt% glucose and 5.9 wt% fructose. Molasses also contained 4.3 g/kg nitrogen, 1.8 g/kg phosphorus, 2.8 g/kg magnesium, 9.5 g/kg potassium and 2.3 g/kg calcium. Commercial FOS (Nutraflora® P-95, Ingredion, Canada) was purchased from a local nutraceutical shop. 1-Kestose, nystose, glucose, fructose, sucrose, and salts were purchased from Sigma-Aldrich (US). 1,1,1-Kestopentaose was purchased from Megazyme (Ireland). All chemicals used in this study were of analytical grade or above.

*Aureobasidium pullulans* FRR 5284, an elite *Aureobasidium* strain identified by the authors (Khatun et al. 2020), was used to produce transfructosylating enzymes. Invertase-deficient *Saccharomyces cerevisiae* 1403-7A was provided by Microbiogen Pty Ltd. Both strains were stored in 30% (v/v) glycerol stock solutions at –80 ℃. Glycerol stocks were streaked onto YPD agar (10 g/L yeast extract, 20 g/L peptone, 20 g/L dextrose and 20 g/L agar) plates, incubated at 28 °C for 3 days. Agar plates were stored at 4 °C prior to use. Single colonies were used for seed culture preparation (*A. pullulans* FRR 5284) or directly used to grow cells for microbial treatment of FOS solutions (*S. cerevisiae* 1403-7A).

Nine probiotic microorganisms (eight *Lactobacillus* strains and one *Bacillus* strain) were used for in vitro prebiotic activity test. *L. arabinosus* QUT 0367, *L. plantarum* QUT 0783, *L. fermentan* QUT 0872, *L. casei* QUT 0873*, L. fermentum* QUT 1057 (ATCC 9338) were sourced from the University of Queensland Microbiology (UQM) culture collection centre; *L. acidophilus* QUT 0953 was sourced from Princess Alexandra Hospital (Brisbane, Australia); *L. fermentum* QUT 0954 was sourced from Royal Perth Hospital (Perth, Australia). *L. fermentum* QUT 0974 was sourced from a commercial probiotics sample. *Bacillus amyloliquefaciens* H57 was a reported probiotic strain (Shini et al. 2020). The strains were stored at -80 °C in 30% (v/v) glycerol and cultured in De Man–Rogosa–Sharpe (MRS) media overnight at 37 °C in anaerobic chambers as required. MRS media contained 20 g/L glucose, 10 g/L peptone, 10 g/L meat extract, 5 g/L yeast extract, 2 g/L K_2_HPO_4_, 1.08 g/L Tween 80, 5 g/L sodium acetate, 2 g/L ammonium citrate (tribasic), 0.2 g/L MgSO_4_, and 0.05 g/L MnSO_4_.

### Transfructosylating enzyme production

#### Preculture

Single colonies of *A. pullulans* FRR 5284 were transferred to preculture medium to prepare inoculum, followed by cultivation at 28 °C and 180 rpm for 2 days (Khatun et al. 2020). After 2 days, the cells were collected by centrifugation at 4000 rpm for 10 min, re-suspended in water and the suspension was clarified by centrifugation. The resulting pellet was resuspended in ultrapure water to a final cell concentration of 2–3 g/L based on dry mass.

#### Production in molasses medium

Transfructosylating enzymes were produced in molasses medium containing a total sugar concentration of 100.0 g/L (sucrose equivalent). Exogenous nitrogen and phosphate (10.0 g/L of NaNO_3_ and 2.2 g/L of Na_2_HPO_4_) were added and the total nitrogen and phosphate concentrations in the molasses media were 2.57 g/L (equivalent to a total of 10 g/L NaNO_3_ and 8.5 g/L yeast extract) and 0.88 g/L (equivalent to 5.0 g/L KH_2_PO_4_), respectively. Additional nutrients and salts were not added to molasses medium because molasses is relatively abundant in micronutrients (e.g., 3.0 g/L MgSO_4_ equivalent, 4.1 g/L KCl equivalent, at 100.0 g/L sugars in molasses). Synthetic molasses medium containing sucrose, glucose, and fructose at the same concentrations as those in molasses was used as a control for enzyme production. The synthetic molasses medium contained total nitrogen and phosphorous concentrations of 2.57 g/L (with addition of 10.0 g/L NaNO_3_ and 8.5 g/L yeast extract) and 0.88 g/L phosphorus (with addition of 5.0 g/L K_2_HPO_4_), respectively, as per the molasses medium. The synthetic molasses medium also contained 0.5 g/L MgSO_4_, which was a commonly used concentration (Dominguez et al. 2012; Shin et al. 2004; Vandáková et al. 2004). Aliquots (45 mL) of molasses and synthetic molasses media were transferred into 250-mL conical flasks and autoclaved at 121 °C for 15 min.

Enzyme production was initiated by inoculation of molasses and synthetic molasses media with 5 mL of well-mixed *A. pullulans* FRR 5284 preculture. The cultures were then incubated at 28 °C and 180 rpm for 120 h. Sub-samples (5 mL) were collected at 24, 48, 72, 96, and 120 h, and centrifuged at 4,000 g for 15 min. An aliquot of supernatant was reserved for measurement of residual sugars, FOS, ethanol, and other metabolites by high-performance liquid chromatography (HPLC), while the remainder was stored at − 20 °C for analysis of extracellular transfructosylating enzymes. The *A. pullulans* FRR 5284 cells in the sub-samples were washed twice with deionized water via centrifugation and freeze-dried for 48 h. The freeze-dried cells were weighed to calculate the cell concentrations. The freeze-dried cells were stored at 4 °C and used in subsequent reactions for enzymatic activity assays. All experiments were conducted in triplicate.

#### Production in synthetic sugar media

To investigate the effect of glucose on transfructosylating enzyme production, five synthetic sugar media containing a total sugar concentration of 100 g/L with the sucrose and glucose mass ratios of 100:0: 90:10; 70:30; 50:50 and 0:100, respectively, were prepared. Medium nutrients and salts were added as per those for synthetic molasses medium. Aliquots (45 mL) of medium were transferred into 250-mL conical flasks and autoclaved at 121 °C for 15 min. The procedures for enzyme production, sampling and sample processing using these media as substrates were the same as those described using molasses media.

### Assays of transfructosylating and hydrolysis activities

Transfructosylating and hydrolysis activities of intracellular and extracellular enzymes were determined based on the methods used in the authors’ previous study (Khatun et al. 2020). Briefly, enzyme activity assay of intracellular enzymes was carried out in 5 mL 50% (w/v) sucrose solutions (in 50 mM sodium acetate buffer, pH 5.5) containing 0.5 g/L freeze-dried cells (using 0.1 mL stock solution containing 25 g/L cells). Activity assay of extracellular enzymes was conducted under the same conditions with the use of 0.1 mL supernatants instead of 0.1 mL of 25 g/L freeze-dried cells. Enzymatic reactions were carried out at 55 ℃ at 100 rpm for 1 h, followed by boiling the reaction mixtures for 15 min to stop the reactions. Sugars and FOS in reaction solutions were determined by HPLC. The monosaccharides produced from the assays were used to calculate the activities of the intracellular and extracellular enzymes. A detailed procedure for the calculation of enzyme activities was described elsewhere (Khatun et al. 2020; Zhang et al. 2016).

### FOS production and microbial treatment

#### FOS production from molasses and sucrose using intracellular transfructosylating enzymes

Sugarcane molasses was diluted to a total sugar concentration of 333 g/L (sucrose equivalent; 255 g/L sucrose, 42 g/L glucose, and 33 g/L fructose) and FOS production was carried out at 55 ℃ and 100 rpm for 12 h in 50 mL solution containing 300 g/L sugars (232 g/L sucrose) and 5 g/L cells. Samples of 5 mL were collected at different time intervals. Sucrose (230 g/L) was used as a control carbon source to produce FOS and the production conditions were the same as those for FOS production using molasses. Small portions of the samples were collected for the determination of sugars and FOS by HPLC analysis.

#### Microbial treatment of molasses FOS

The invertase-deficient *Saccharomyces cerevisiae* was used to consume monosaccharides (glucose and fructose) in molasses FOS. The yeast cell was firstly prepared in a 500-mL shake flask containing 350 mL YPD for 8 h at 30 °C and 180 rpm. After centrifuging the fermentation medium at 4,000 rpm for 15 min, the cell biomass was resuspended in sterile water after being rinsed twice with sterile water. For monosaccharide removal, molasses FOS of 30 mL was aerobically incubated with 15 g/L *Saccharomyces* yeast cells at 35 ºC for 9 h at 180 rpm. After incubation, the yeast cells were removed by centrifugation. Small portions of supernatants were analyzed by HPLC to determine the concentrations of sugars, metabolites and the rest of the supernatants were collected for prebiotic activity assay.

### Prebiotic activity test

For prebiotic activity assay, MRS media were used with the replacement of 20 g/L glucose with 10 g/L FOS or non-FOS sugars. MRS media without sugars were firstly autoclaved at 121 ℃ for 15 min, followed by mixing with FOS and non-FOS sugar samples pre-sterilized by filtration. The FOS samples included two FOS samples generated from molasses (3 and 9 h FOS production) and treated by the invertase-deficient yeast, two FOS samples generated from sucrose (3 and 9 h FOS production) and one commercial FOS (Nutraflora P-95). The non-FOS sugar samples included one glucose, one sucrose, one sugarcane molasses, and one synthetic molasses. The concentrations of total sugars (FOS and non-FOS sugars) in each medium were 10 g/L for prebiotic activity test.

Overnight activated and sub-cultured probiotic strains were centrifuged, washed, and suspended in water for inoculation. To start the prebiotic test, 15-mL tubes containing 13 mL of MRS media with various carbon sources at a concentration of 10 g/L were inoculated with 1 mL of probiotic strain solution, which was equivalent to an initial probiotic OD_600_ value of 0.02. The lid-on tubes were incubated in an anaerobic chamber at 37 °C for 48 h. Samples were withdrawn at different time intervals to measure the optical densities at 600 nm (OD_600_) using GloMax® discover microplate reader (Promega, Australia). The background OD_600_ values were subtracted by measuring the optimal densities of cell-free media after centrifugation. The OD_600_ values of all *Lactobacillus* and *Bacillus* strains growing in the media containing FOS generated in this study and non-FOS sugar were compared with those in the medium containing the commercial FOS (NutraFlora P95) to calculate the cell growth improvements.

### Analytic methods

#### Determination of sugars and FOS by HPLC

Sugars (glucose, fructose and sucrose) and FOS in molasses, cultivation media and FOS production solutions were determined by an HPLC system. The system was equipped with an Asahipak NH2P-50 4E column (Shodex, Tokyo, Japan) and RI detector. The column temperature was 27 °C with the use of 70% (v/v) acetonitrile as a mobile phase at a flow rate of 1.0 mL/min. The detailed method was described in the authors’ previous publication (Khatun et al. 2020).

#### Elemental analysis of molasses

Molasses nitrogen in molasses was determined by a TOC/TN analyzer (Shimadzu, Japan) while other elements were determined by an inductively coupled plasma optical emission spectrometer (ICP-OES) (Thermo Fisher Scientific). Detailed analysis methods were available elsewhere in the author’s previous study (Khatun et al. 2021).

### Statistical analysis

Unless otherwise mentioned, all the experiments in this study were conducted in triplicate. Student's t-test was used for statistical analysis. A significance level for all statistical tests was considered at *p* < 0.05. The data were presented as the mean values of triplicate with standard deviations.

## Results and discussion

### Transfructosylating enzyme production from molasses

**Fig. 1:**
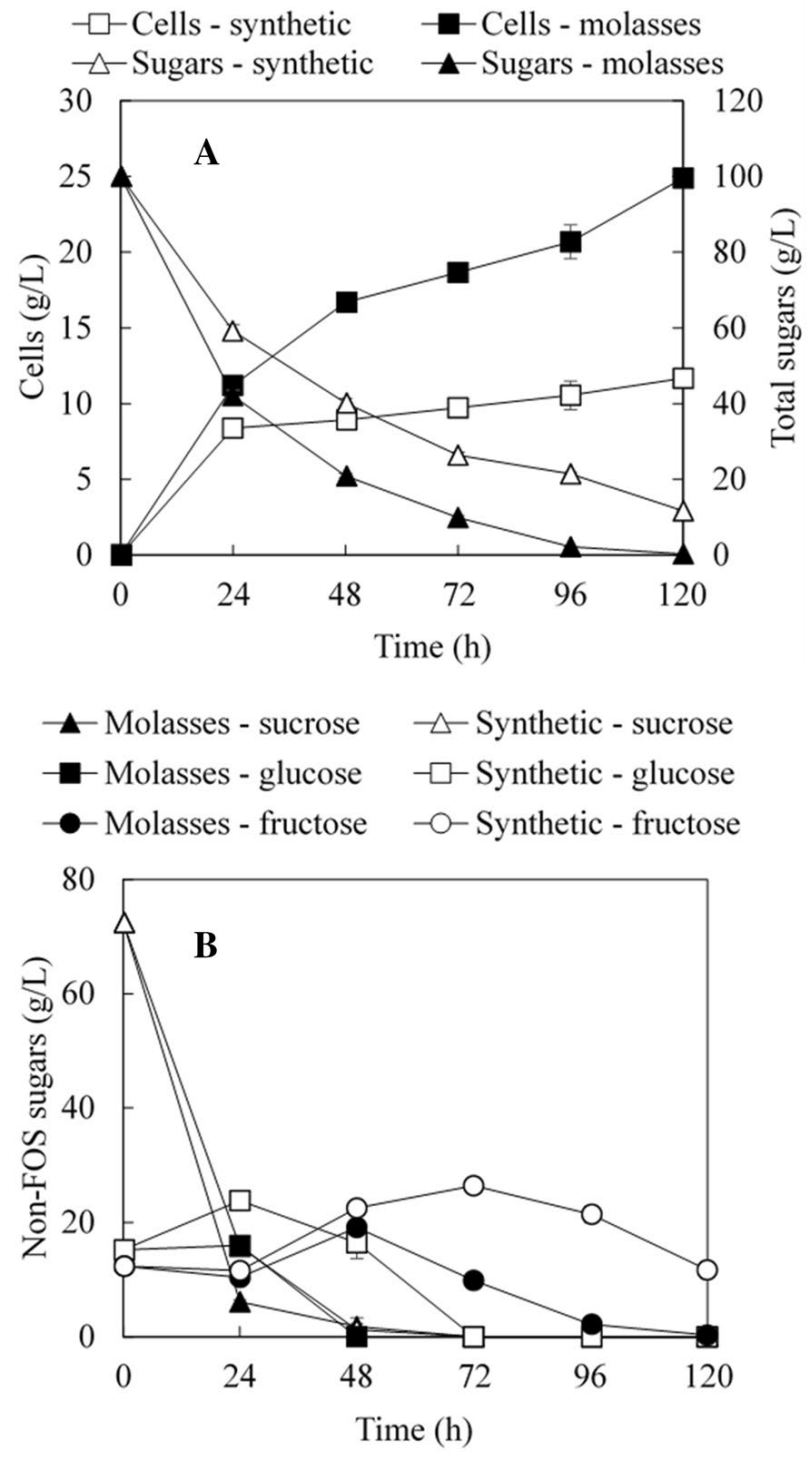
Kinetics of **A** cell production and total sugar consumption, and **B** individual non-FOS sugars using molasses and synthetic molasses media by *A. pullulans* FRR 5284

Molasses is a nutrient-rich sugar source. In this study, molasses was used to produce transfructosylating enzymes by *A. pullulans* FRR 5284 with addition of only two exogenous nitrogen and phosphorous sources (NaNO_3_ and Na_2_HPO_4_). Figure 1A shows cell production and total sugar consumption with molasses medium and synthetic molasses medium. *A. pullulans* FRR 5284 grew faster in molasses medium and sugar consumption rate was also higher than that in synthetic molasses medium. Furthermore, Fig. 1B shows the concentration changes of sucrose, glucose and fructose in cultivation. Overall, the consumption of these sugars in molasses medium was faster than that in synthetic molasses medium. Sucrose was almost depleted at 48 h in both media due to the conversion of sucrose to glucose and fructose as well as the synthesis of small amounts of FOS (data not shown). Glucose was consumed more rapidly by *A. pullulans* FRR 5284 than fructose, consistent with the preference for glucose observed for most microorganisms. The concentration of fructose increased in the middle of the cultivation because of the hydrolysis of sucrose and slow consumption by the strain.

**Fig. 2:**
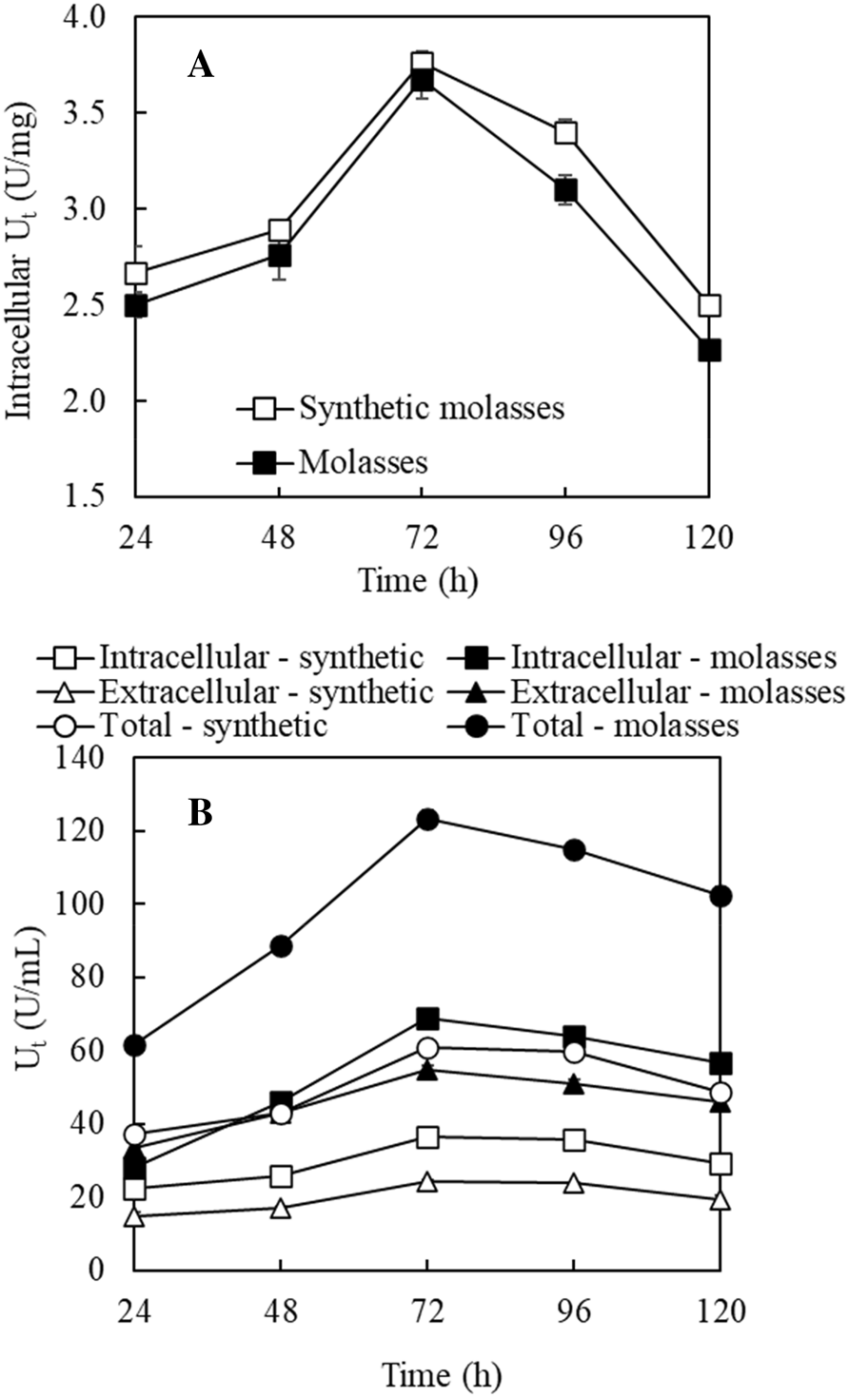
Transfructosylating activities (U_t_) of enzymes produced using molasses and synthetic molasses media by *A. pullulans* FRR 5284. **A** Intracellular activities based on cell mass; **B** intracellular and extracellular activities based on medium volume

Regarding intracellular transfructosylating enzyme production, as shown in Fig. 2A, the use of synthetic molasses medium led to higher U_t_ (U/mg cells) based on cell mass from 24 to 120 h with the U_t_ peaked at 72 h. Figure 2B shows intracellular, extracellular, and total U_t_ (U/mL) based on cultivation volume. Overall, the use of sugarcane molasses medium led to higher U_t_ (U/mL) than the use of synthetic medium due to the production of higher concentrations of cell mass using the sugarcane molasses medium. The highest total U_t_ (123.6 U/mL) was achieved at 72 h, corresponding to the highest intracellular and extracellular U_t_ of 68.7 U/mL and 54.9 U/mL, respectively. Higher intracellular U_t_ is preferred as intracellular enzymes can be harvested readily by filtration or centrifugation. The collected cells may be used directly for FOS production. Surprisingly, U_h_ was not detected in either cells or fermentation broth when sugarcane molasses or synthetic molasses medium was used.

Faster cell growth in sugarcane molasses-based medium than that in synthetic molasses medium was attributed to the abundance and diversity of nutrients in sugarcane molasses (Dorta et al. 2006). The rapid reduction of intracellular U_t_ (U/mg cells) after 72 h cultivation was likely due to the reduced transfructosylating enzyme production because of the depletion of sucrose (Fig. 1B), which is an inducer for producing inducible transfructosylating enzymes. The activity (U/g cells) reduction may also be attributed to the dilution effect due to the increased cell concentration. The slow reduction of total U_t_ (U/mL) based on medium volume after 72 h cultivation was mainly due to the increased cell concentrations offsetting the rapid decrease of U_t_ (U/mg cells). It is worth mentioning that U_h_ was not detected with the use of molasses-based and synthetic molasses-based media, indicating a relatively low level of U_h_. It is expected that the low U_h_ favors the production of FOS as hydrolysis of produced FOS will be slow.

A previous study showed that with the cultivation of *A. pullulans* DSM 2404 in a medium containing 50 g/L sucrose, the total U_t_ (U/flask) of one individual enzyme increased in the first 2 days and then decreased while the activities of all the other four individual enzymes continue to increase during the 3-day cultivation period (Yoshikawa et al. 2006). In another study with the cultivation of *A. pullulans* KCCM 12017 in a sucrose medium containing 100 g/L sucrose, both intracellular and extracellular total U_t_ (U/mL) increased in 3-day cultivation (Shin et al. 2004). In the present study, both intracellular and extracellular U_t_ (both U/mg cells and U/mL) increased first, and then decreased after 72 h cultivation. These different observations indicate that the U_t_ (U/volume) change during cultivation was affected by cell concentrations and U_t_ (U/mg cells). Moreover, since the transfructosylating enzymes were likely composed of mixed enzymes (Salinas and Perotti 2009; Shin et al. 2004; Vandáková et al. 2004; Xie et al. 2017; Yoshikawa et al. 2006; Zhang et al. 2016), measured U_t_ (U/mg cells) was likely the overall activity of total enzymes rather than individual enzymes.

### Effect of sucrose/glucose mass ratio on enzyme production

Since transfructosylating enzymes produced by microorganisms likely included both constitutive and inducible enzymes and molasses contained mixture sugars, such as sucrose (an inducer for inducible transfructosylating enzymes) and mono-sugars (glucose and fructose), further study was carried out to understand the effect of different carbon source combinations on the production of transfructosylating enzymes.

**Fig. 3:**
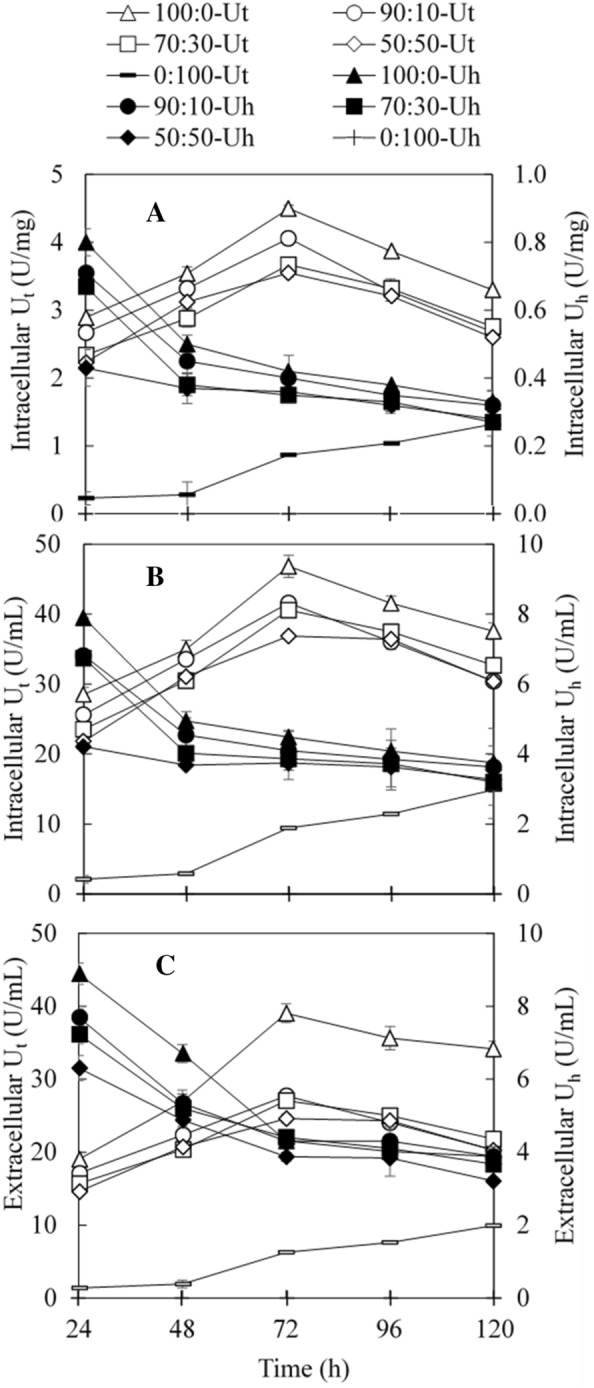
Effect of sucrose/glucose mass ratio on transfructosylating enzyme production by *A. pullulans* FRR 5284. **A** Intracellular transfructosylating (U_t_) and hydrolytic (U_h_) activities based on cell mass; **B** intracellular U_t_ and U_h_ based on cultivation volume; **C** extracellular U_t_ and U_h_ based on cultivation volume

Figure 3 shows the effect of the sucrose/glucose mass ratio in synthetic sugar media on U_t_ and U_h_ generated by *A. pullulans* FRR 5284. The highest U_t_ values (both U/mg cells and U/mL medium) were achieved at 72 h with the use of pure sucrose medium while the inclusion of glucose at the mass ratio from 100:0 to 50:50 in the medium reduced the maximum U_t_. With the use of pure glucose medium, the U_t_ was very low in the beginning of the monitored cultivation period (from 24 to 120 h) and increased gradually with the prolonged cultivation time. The maximum U_t_ with pure glucose medium was achieved at the end of the cultivation (120 h), which was much lower than that with the media having sucrose/glucose mass ratios of 100:0 – 50:50.

The use of pure sucrose medium also led to the highest U_h_ values (both U/mg cells and U/mL medium) though the maximum values were observed at the beginning of the monitored cultivation period (from 24 to 120 h). Inclusion of glucose in the medium reduced U_h_ and the trend was similar to that observed for U_t_. Differently from U_t_, U_h_ continued to decrease from 24 to 120 h in the media containing sucrose/glucose mass ratios of 100:0 – 50:50. With the use of pure glucose medium, U_h_ was not detected in the monitored cultivation period (from 24 to 120 h). It was noted that U_h_ was not detected in the sucrose-rich molasses and synthetic molasses media though both media contained sucrose, indicating the presence of fructose may also affect the enzyme production.

The above results indicated that transfructosylating enzymes produced by *A. pullulans* FRR 5284 included both constitutive and inducible enzymes while constitutive enzymes were likely the dominant ones. In the presence of sucrose, both constitutive and inducible enzymes were expressed while in the glucose-based medium, only constitutive enzymes were produced with very low U_t_ (Fig. 3). The observations in the present study were in line with previous results in the literature which reported the production of constitutive enzymes in the presence of glucose (Nascimento et al. 2019; Yoshikawa et al. 2006, 2008).

### FOS production from sugarcane molasses using *A. pullulans* cells

#### FOS production

Following production of transfructosylating enzymes using sugarcane molasses medium, FOS were produced from sugarcane molasses by *A. pullulans* cells, which contained intracellular transfructosylating enzymes. Although FOS could be produced from high concentrations of sucrose solutions containing 800 g/L sucrose (Zhang et al. 2016), original sugarcane molasses (containing 41 wt% sucrose) was very viscous and had to be diluted to a solution containing 230 g/L sucrose. *A. pullulans* cells were directly used for FOS production as the cells were easy to collect and recycle. In addition, cells could be freeze-dried for long-term storage.

**Fig. 4:**
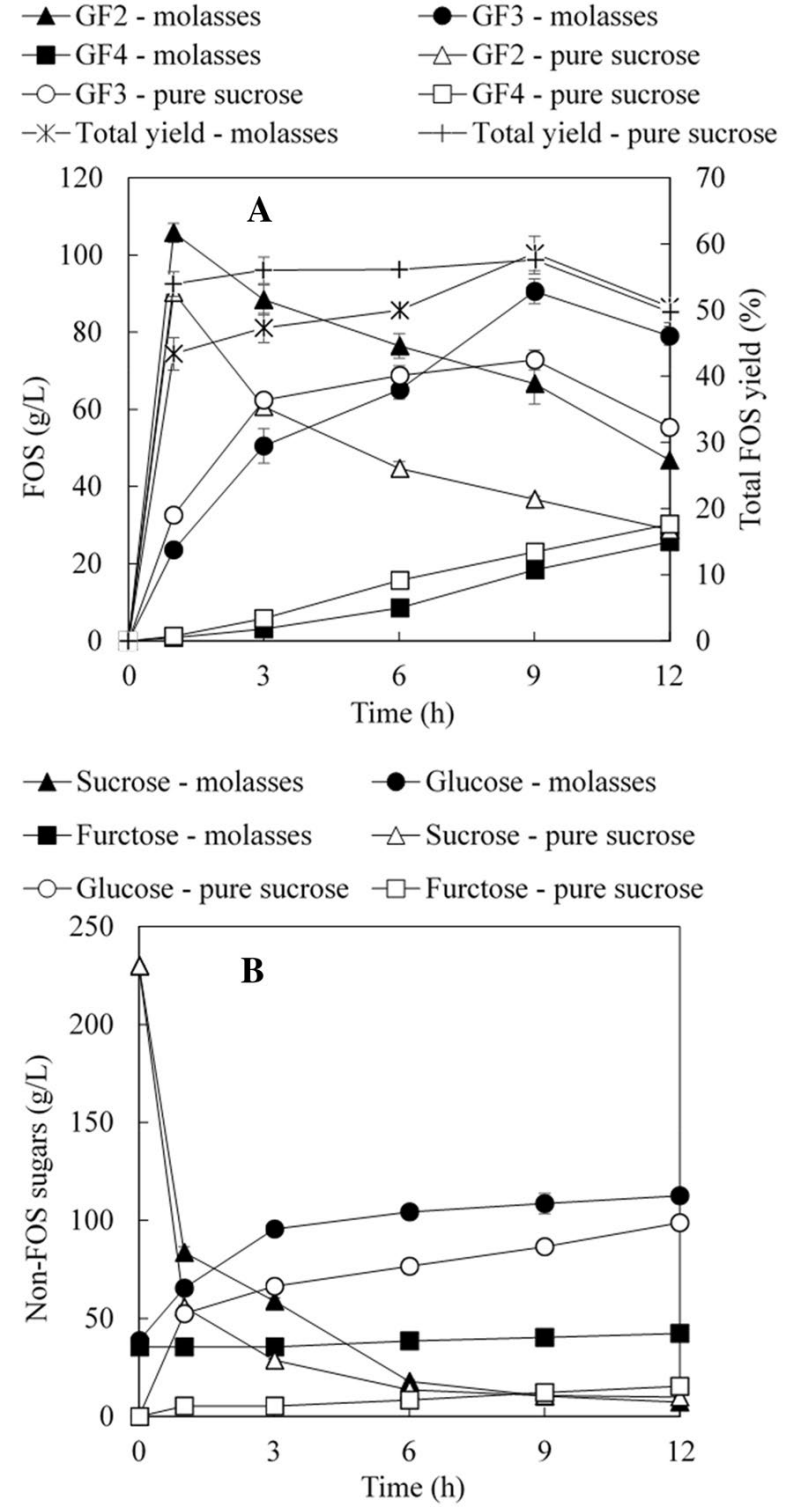
Kinetics of FOS production using sugarcane molasses and sucrose by *A. pullulans* cells. **A** Individual FOS concentration and total FOS yields; **B** non-FOS sugars

Figure 4A shows the kinetics of FOS production and the total FOS yields in the production process. For comparison, pure sucrose was included as a control carbon source. As shown in Fig. 4A, FOS production was very rapid and the total FOS yields reached 43–54% within 1 h. The use of molasses led to a lower FOS yield of 43%, which was mainly attributed to the inhibition from existing glucose (Khatun et al. 2020). With sucrose as the substrate, the total FOS yield reached a plateau (56–58%) after 3 h reaction while with molasses, the total FOS yield gradually increased to the highest value of ~ 59% at 9 h. The pure sucrose substrate also led to higher individual FOS concentrations than sugarcane molasses except for the higher GF_3_ concentration with molasses at 9 h and 12 h. In addition, GF_2_ was rapidly produced within 1 h and the concentrations of GF_2_ started to reduce after 1 h while the concentration of GF_3_ increased in the first 9 h and started to decrease after 9 h reaction. In contrast, the concentrations of GF_3_ increased steadily in the reaction time. The kinetics of FOS production was in line with previous studies (Sangeetha et al. 2004; Shin et al. 2004; Zhang et al. 2016, 2019). Along with FOS production, significant amounts of glucose and fructose were accumulated and FOS from molasses contained much higher levels of glucose and fructose (Fig. 4B).

#### FOS content improvement by microbial treatment

Prebiotics such as FOS need to resist the gastric acidity, hydrolysis by mammalian enzymes and gastrointestinal absorption before it can reach intestinal microflora (Gibson et al. 2004). Glucose is the major by-product in FOS solutions and the presence of glucose will reduce the FOS content and delivery efficiency of FOS. In order to improve FOS content in the produced FOS solutions, various approaches have been used to improve FOS content and purity following FOS production, which include expensive ultra-filtration and chromatographic separation technologies (Nobre et al. 2014, 2012; Pinelo et al. 2009). In addition, microbial treatment was also used to improve FOS content (Nobre et al. 2016; Yang et al. 2008). Microbial treatment could not lead to the production of high-purity FOS, but the resultant FOS mixtures may be qualified for feed application. *S. cerevisiae* (invertase-deficient yeast) is able to convert monosaccharides to ethanol, reducing the contents of digestible sugars and improving the purity of non-digestible FOS.

**Fig. 5:**
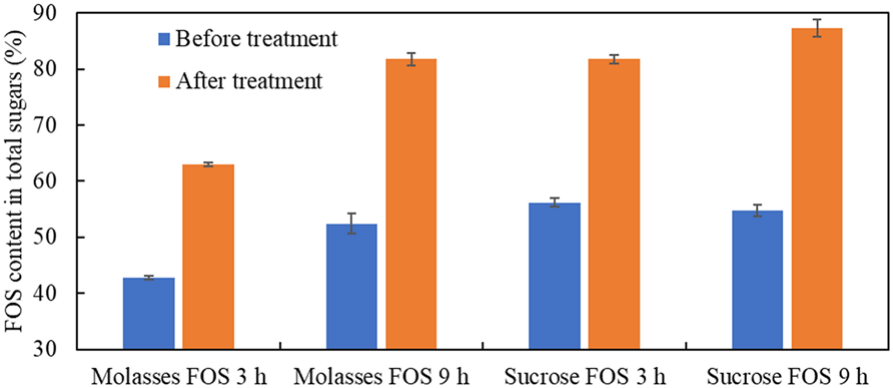
FOS content improvement after treatment by an invertase-deficient yeast

In the present study, FOS samples (3 and 9 h reaction times) produced from sugarcane molasses and pure sucrose were further treated with an invertase-deficient yeast. Following treatment, glucose in FOS solutions were completely removed and fructose was still present in some samples though the concentrations were reduced while sucrose concentrations remained unchanged (data not shown). Figure 5 shows the changes of FOS contents in total sugars after treatment. FOS contents were improved to 63.0–87.0% after treatment, ~ 20–30% higher than corresponding contents prior to treatment.

Table 1 shows the FOS profiles after treatment. Treated FOS samples did not contain glucose and had varying GF_2_/GF_3_/GF_4_ ratios. After treatment by the invertase-deficient *S. cerevisiae* yeast, ethanol and acetic acid were produced. Interestingly, lower concentrations of ethanol and acetic acid were produced from FOS solutions derived from molasses, which had higher glucose concentrations than the corresponding FOS solutions derived from pure sucrose. Under aerobic conditions, the metabolic activities of yeast in the FOS solution derived from molasses were likely very intensive, leading to the generation of large amounts of CO_2_ and yeast cell mass. In contrast, the FOS solutions derived from pure sucrose solution had little nutrients and the added yeast cells were more like enzyme carriers, which produced ethanol and acetic acid as the main products with the generation of fewer amounts of CO_2_ and yeast cell mass.

**Table 1:** FOS and non-FOS sugar ratios after treatment by invertase-deficient yeast

Component	Molasses			Sucrose		Nutraflora P95
	3 h	9 h		3 h	9 h	
GF_2_^a^	1.00	1.00		1.00	1.00	1.00
GF_3_	0.57	1.36		1.03	1.98	1.47
GF_4_	0.04	0.28		0.10	0.63	0.20
Fructose	0.28	0.43		0.00	0.23	–^b^
Sucrose	0.67	0.16		0.47	0.29	–^b^

^a^The concentrations of GF_3_, GF_4_, fructose and sucrose were compared to the concentrations of GF_2_

^b^Glucose, fructose and sucrose were not detected in Nutraflora P95

Previously, a two-step fermentation process by using *S. cerevisiae* and *A. pullulans* strains improved FOS purity from 51.7 to 81.6%. However, the process needs to be carefully controlled as the *S. cerevisiae* strain was not invertase-deficient and could consume FOS (Nobre et al. 2016, 2012). In another study, a sequential FOS production and purification was carried with the use of the *Aspergillus japonicus* strain (FOS producer) and *Pichia pastoris* (reducing sugar consumer). The *Pichia pastoris* strain converted glucose to glycerol and improved FOS content from 56.6 to 84.4% (Yang et al. 2008). The removal of glycerol from FOS solution still needs the use of expensive chromatographic technology if the FOS is produced for human consumption. In the present study, glucose was converted to ethanol and acetic acid by the invertase-deficient *S. cerevisiae* yeast. The ethanol and organic acid-containing FOS may be directly used as feed prebiotics and the presence of ethanol and acetic acid may help to prevent microbial contamination.

### Prebiotic activities of treated FOS

For feed application, further removal of impurities in molasses FOS solutions after microbial treatment may not be necessary if the semi-purified FOS solutions have prebiotic activities since molasses is already a commercial feed supplement. Therefore, in vitro prebiotic activity test was carried out to check the prebiotic activities of the semi-purified FOS solutions. A total of nine probiotic strains, including eight *Lactobacillus* strains and one *Bacillus* strain were used for in vitro prebiotic activity test. In addition to the FOS samples produced in this study, a commercial FOS (Nutraflora P95) and several non-FOS sugars were also included for comparison. Table 2 shows the compositions of the FOS and non-FOS sugars used for prebiotic activity test at 10 g/L. These FOS and non-FOS sugars used for prebiotic assays had different sugar profiles.

**Table 2:** Compositions of FOS sugars and non-FOS sugars for in vitro prebiotic activity assay

Component (g/L)	Molasses FOS—3 h	Molasses FOS—9 h	Sucrose FOS—3 h	Sucrose FOS—9 h	Commercial FOS	Molasses	Synthetic molasses	Glucose	Sucrose
GF2	3.9 ± 0.6	3.1 ± 0.4	3.9 ± 0.1	2.4 ± 0.3	3.6 ± 0.2	–	–	–	–
GF3	2.2 ± 0.3	4.2 ± 0.4	4.0 ± 0.2	4.8 ± 0.1	5.3 ± 0.1	–	–	–	–
GF4	0.1 ± 0.0	0.9 ± 0.1	0.4 ± 0.0	1.5 ± 0.1	1.1 ± 0.1	–	–	–	–
Fructose	1.1 ± 0.3	1.3 ± 0.1	-	0.6 ± 0.0	–	1.1	1.1	–	–
Glucose	–	–	–	–	–	1.3	1.3	10.0	–
Sucrose	2.6 ± 0.4	0.5 ± 0.1	1.8 ± 0.2	0.7 ± 0.1	–	7.7	7.7	–	10.0
Total non-FOS sugars	3.7	1.8	1.8	1.3	–	–	–	–	–
Total FOS	6.3	8.2	8.2	8.7	10.0	–	–	–	–
Total sugar	10.0	10.0	10.0	10.0	10.0	10.0	10.0	10.0	10.0
Ethanol	1.6 ± 0.2	1.9 ± 0.3	1.5 ± 0.1	2.6 ± 0.4	–	–	–	–	–
Acetic acid	0.2 ± 0.0	0.4 ± 0.0	0.5 ± 0.1	0.6 ± 0.1	–	–	–	–	–
Total metabolites	1.8	2.3	2.0	3.2	–	–	–	–	–

**Fig. 6:**
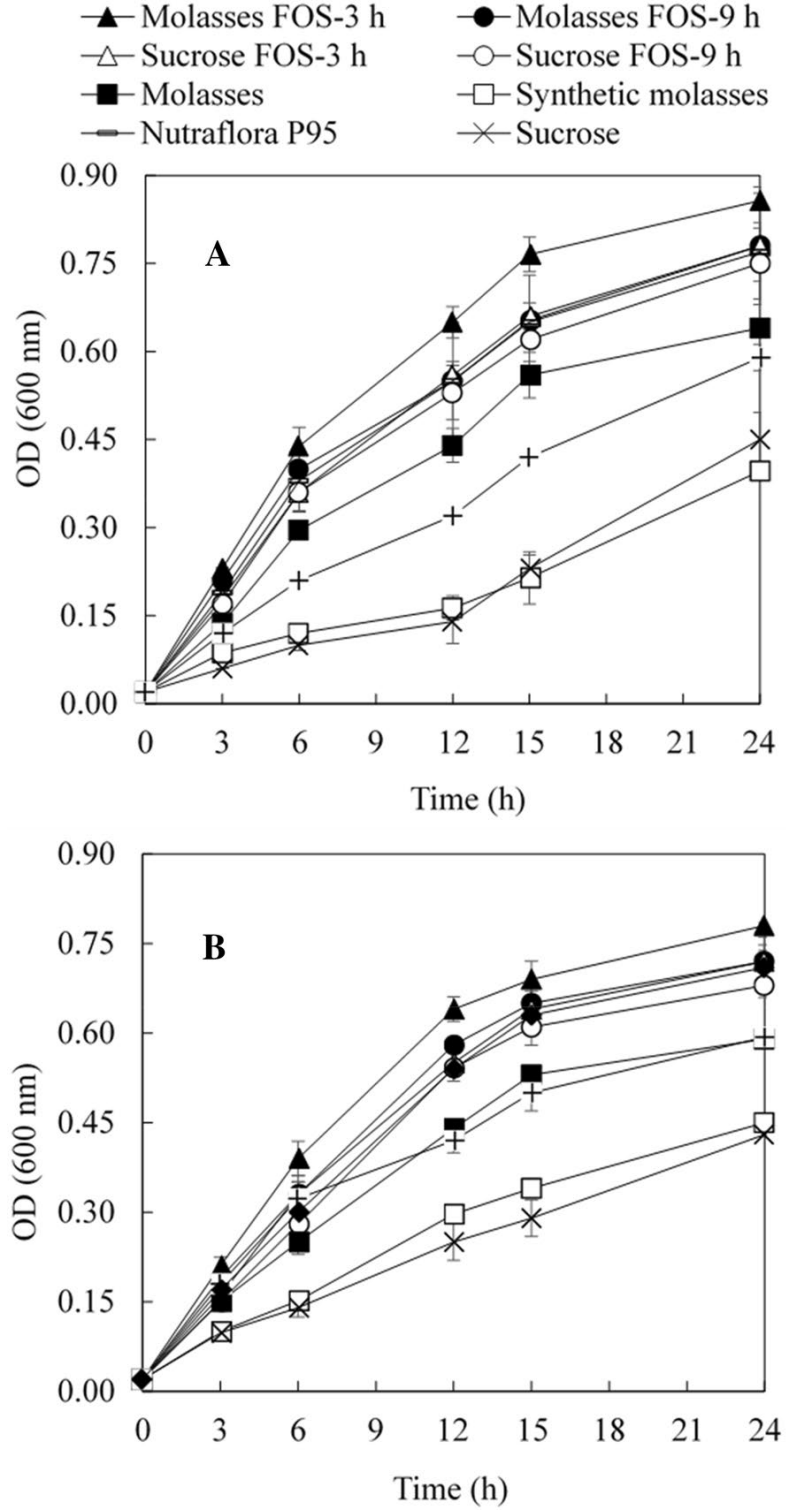
Representative growth curves of two probiotics in the presence FOS and non-FOS sugars. **A** *Bacillus* H 57, **B** *L. casei* QUT 0873

Figure 6 shows the anaerobic growth (as indicated by OD_600_ value) of two representative probiotic strains in the presence of FOS and non-FOS sugars with the growth of all the nine strains after 12 h and 24 h incubation summarized in Additional file 1: Table S1. Furthermore, the growth improvements of probiotic strains with different carbon sources are summarized in Table 3 in comparison with the commercial FOS (Nutraflora P95). Overall, the use of treated 3-h molasses FOS led to the most significant growth improvements of all the probiotics, followed by the use of treated 9-h molasses FOS. The use of treated 3-h sucrose FOS led to slight growth improvements of most tested probiotics while the use of treated 9-h sucrose FOS resulted in the reduced growth of most tested probiotics compared to the use of Nutraflora P95. Regarding the non-FOS sugars, the use of sucrose resulted in the most significant reductions of most tested probiotics, followed by synthetic molasses and sugarcane molasses. Interestingly, the use of glucose also led to significant growth reductions of most tested probiotics. These results indicated that FOS rather than non-FOS sugars were preferred by most of the tested probiotics, which justified the need to improve FOS content.

**Table 3:** Cell growth improvements (%) of probiotics compared with the commercial FOS (Nutraflora P95)

Incubation time—strain	Molasses FOS—3 h	Molasses FOS—9 h	Sucrose FOS—3 h	Sucrose FOS—9 h	Molasses	Synthetic molasses	Glucose	Sucrose
12 h—*L. arabinosus* QUT 0367	9.3	1.9	0.0	− 9.3	− 24.1	− 57.4	− 40.7	− 68.5
12 h—*L. plantarum* QUT 0783	17.9	5.4	5.4	− 1.8	− 33.9	− 53.6	− 21.4	− 69.6
12 h—*L. fermentan* QUT 0872	30.9	3.6	7.3	− 5.5	− 21.8	− 47.3	− 12.7	− 43.6
12 h—*L. casei* QUT 0873	18.5	7.4	1.9	0.0	− 18.5	− 44.4	− 22.2	− 53.7
12 h—*L. acidophilus* QUT 0953	45.5	9.1	9.1	− 11.4	− 9.1	− 40.9	− 4.5	− 50.0
12 h—*L. fermentum* QUT 0954	62.9	8.6	2.9	2.9	0.0	− 28.6	− 8.6	− 57.1
12 h—*L. fermentum* QUT 0974	37.2	11.6	0.0	− 9.3	− 34.9	− 53.5	0.0	− 48.8
12 h—*L. fermentum* QUT 1057	28.2	4.2	-8.5	− 21.1	− 36.6	− 47.9	− 7.0	− 53.5
12 h—*Bacillus* H57	18.2	0.0	1.8	− 3.6	− 20.0	− 70.9	− 41.8	− 74.5
24 h—*L. arabinosus* QUT 0367	18.5	6.2	3.1	0.0	− 15.4	− 41.5	− 12.3	− 46.2
24 h—*L. plantarum* QUT 0783	9.7	0.0	0.0	− 6.9	− 18.1	− 25.0	− 19.4	− 55.6
24 h—*L. fermentan* QUT 0872	21.7	2.9	4.3	− 8.7	− 20.3	− 36.2	− 13.0	− 29.0
24 h—*L. casei* QUT 0873	9.9	1.4	1.4	− 4.2	− 16.9	− 36.6	− 16.9	− 39.4
24 h—*L. acidophilus* QUT 0953	14.9	-1.5	1.5	− 7.5	− 29.9	− 35.8	− 7.5	− 35.8
24 h—*L. fermentum* QUT 0954	24.6	4.6	0.0	− 4.6	− 32.3	− 32.3	− 10.8	− 47.7
24 h—*L. fermentum* QUT 0974	11.1	1.6	3.2	− 7.9	− 36.5	− 38.1	− 7.9	− 42.9
24 h—*L. fermentum* QUT 1057	18.8	2.5	-1.3	− 15.0	− 26.3	− 31.3	− 8.8	− 21.3
24 h—*Bacillus* H57	11.7	1.3	1.3	− 2.6	− 16.9	− 50.6	− 23.4	− 41.6

In vitro assays are often carried out to assess the prebiotic activities of FOS and other oligosaccharides. In some studies, glucose was included as a reference sugar, which led to either superior, inferior, or comparable growth of tested probiotics to prebiotic oligosaccharides (Huebner et al. 2007; Nobre et al. 2019, 2018). In the present study, the use of glucose led to reduced growth of most tested probiotics. However, it should be noted that the comparison of the growth of probiotics with FOS and non-FOS sugars only indicates the carbon source preference by the probiotics. The better growth with non-FOS sugars, such as glucose does not mean that these non-FOS sugars are prebiotics. In the real animal digestion system, non-FOS sugars, especially glucose, can be readily absorbed and utilized before they reach the intestinal microflora/probiotics. Furthermore, in vivo prebiotic activity assay is also needed in future studies to verify the role and benefits of molasses FOS in improving animal health and promoting animal production.

## Conclusions

This study demonstrated a simple process to produce transfructosylating enzymes by one *A. pullulans* strain and FOS from molasses using whole-cell biocatalyst. *A. pullulans* in molasses medium produced higher U_t_ than those in synthetic molasses or pure sucrose medium. Glucose in molasses inhibited U_h_, favoring FOS production. A simple microbial treatment using invertase-deficient yeast enhanced the FOS content with converting glucose to ethanol and acetic acid. One treated FOS sample derived from molasses led to higher in vitro prebiotic activities with the nine probiotic strains, indicating the commercial potential of molasses FOS as a valuable feed supplement.

### Supplementary Information

**Additional file 1**: **Table S1**. Anaerobic growth (indicated by OD600 values) of nine probiotic strains in the presence of FOS and non-FOS sugars.

## Data Availability

The data and the materials are all available in this article and the supporting information.

## Abbreviations

*A. pullulans*: *Aureobasidium pullulans*
FOS: Fructooligosaccharides
GF_2_: Kestose
GF_3_: Nystose
GF_4_: 1,1,1-Kestopentaose
U_t_: Transfructosylating activity
U_h_: Hydrolysis activity
