# Supplementary material for: Transformation of sugarcane molasses into fructooligosaccharides with enhanced prebiotic activity using whole-cell biocatalysts from Aureobasidium pullulans FRR 5284 and an invertase-deficient Saccharomyces cerevisiae 1403-7A
Source: Bioresour Bioprocess. 2021 Sep 3;8(1):85. doi: 10.1186/s40643-021-00438-7 (PMC10992603; doi:10.1186/s40643-021-00438-7)
Supplement: Supplementary file 1 — Additional file 1: Table S1. Anaerobic growth (indicated by OD600 values) of nine probiotic strains in the presence of FOS and non-FOS sugars. [file 40643_2021_438_MOESM1_ESM.docx]

**Transformation of sugarcane molasses into fructooligosaccharides with enhanced prebiotic activity using whole-cell biocatalysts from *Aureobasidium pullulans* FRR 5284 and an invertase-deficient *Saccharomyces* *cerevisiae* 1403-7A**

Most Sheauly Khatun ^1,2^, Morteza Hassanpour ^1,2^, Solange I. Mussatto ^3^, Mark D. Harrison ^1,4^, Robert E. Speight ^1,4,5^, Ian M. O’Hara ^1,2,5^, Zhanying Zhang ^1,2,5*^

^1^ Centre for Agriculture and the Bioeconomy, Faculty of Science, Queensland University of Technology, Brisbane, QLD 4000, Australia.

^2^ School of Mechanical, Medical and Process Engineering, Faculty of Engineering, Queensland University of Technology, Brisbane, QLD 4000, Australia.

^3^ Department of Biotechnology and Biomedicine, Technical University of Denmark, Søltofts Plads, Building 223, 2800, Kongens Lyngby, Denmark.

^4^ School of Biology and Environmental Science, Faculty of Science, Queensland University of Technology, Brisbane, QLD 4000, Australia.

^5^ ARC Centre of Excellence in Synthetic Biology, QUT, Brisbane, QLD 4000, Australia

* Corresponding author, Dr Zhanying Zhang

Email: [jan.zhang@qut.edu.au](mailto:jan.zhang@qut.edu.au)

**Table S1** Anaerobic growth (indicated by OD_600_ values) of nine probiotic strains in the presence of FOS and non-FOS sugars

| Incubation time - Strain | Molasses FOS-3 h | Molasses FOS-9 h | Sucrose FOS-3 h | Sucrose FOS-9 h | Nutraflora P95 | Molasses | Synthetic molasses | Glucose | Sucrose |
| --- | --- | --- | --- | --- | --- | --- | --- | --- | --- |
| 12 h *- L. arabinosus* QUT 0367 | 0.59±0.03 | 0.55±0.03 | 0.54±0.03 | 0.49±0.02 | 0.54±0.01 | 0.41±0.03 | 0.23±0.03 | 0.32±0.02 | 0.17±0.00 |
| 12 h *- L. plantarum* QUT 0783 | 0.66±0.01 | 0.59±0.00 | 0.59±0.01 | 0.55±0.01 | 0.56±0.01 | 0.37±0.02 | 0.26±0.01 | 0.44±0.01 | 0.17±0.02 |
| 12 h *- L. fermentans* QUT 0872 | 0.72±0.01 | 0.57±0.00 | 0.59±0.03 | 0.52±0.03 | 0.55±0.03 | 0.43±0.03 | 0.29±0.00 | 0.48±0.02 | 0.31±0.01 |
| 12 h *- L. casei* QUT 0873 | 0.64±0.02 | 0.58±0.01 | 0.55±0.03 | 0.54±0.01 | 0.54±0.01 | 0.44±0.00 | 0.30±0.01 | 0.42±0.01 | 0.25±0.00 |
| 12 h *- L. acidophilus* QUT 0953 | 0.64±0.02 | 0.48±0.00 | 0.48±0.02 | 0.39±0.03 | 0.44±0.05 | 0.40±0.00 | 0.26±0.03 | 0.42±0.03 | 0.22±0.01 |
| 12 h *- L. fermentum* QUT 0954 | 0.57±0.03 | 0.38±0.03 | 0.36±0.03 | 0.36±0.02 | 0.35±0.06 | 0.35±0.03 | 0.25±0.03 | 0.32±0.01 | 0.15±0.00 |
| 12 h *- L. fermentum* QUT 0974 | 0.59±0.00 | 0.48±0.02 | 0.43±0.00 | 0.39±0.02 | 0.43±0.07 | 0.28±0.02 | 0.20±0.01 | 0.43±0.00 | 0.22±0.01 |
| 12 h *- L. fermentum* QUT 1057 | 0.91±0.02 | 0.74±0.00 | 0.65±0.02 | 0.56±0.03 | 0.71±0.05 | 0.45±0.00 | 0.37±0.03 | 0.66±0.03 | 0.33±0.01 |
| 12 h *- Bacillus* H57 | 0.65±0.03 | 0.55±0.00 | 0.56±0.02 | 0.53±0.05 | 0.55±0.10 | 0.44±0.03 | 0.16±0.02 | 0.32±0.00 | 0.14±0.04 |
| 24 h *- L. arabinosus* QUT 0367 | 0.77±0.03 | 0.69±0.02 | 0.67±0.02 | 0.65±0.02 | 0.65±0.10 | 0.55±0.02 | 0.38±0.02 | 0.57±0.02 | 0.35±0.02 |
| 24 h *- L. plantarum* QUT 0783 | 0.79±0.02 | 0.72±0.01 | 0.72±0.02 | 0.67±0.02 | 0.72±0.00 | 0.59±0.02 | 0.54±0.01 | 0.58±0.01 | 0.32±0.02 |
| 24 h *- L. fermentans* QUT 0872 | 0.84±0.02 | 0.71±0.02 | 0.72±0.03 | 0.63±0.02 | 0.69±0.02 | 0.55±0.03 | 0.44±0.01 | 0.60±0.03 | 0.49±0.03 |
| 24 h *- L. casei* QUT 0873 | 0.78±0.01 | 0.72±0.04 | 0.72±0.03 | 0.68±0.02 | 0.71±0.03 | 0.59±0.01 | 0.45±0.02 | 0.59±0.01 | 0.43±0.00 |
| 24 h *- L. acidophilus* QUT 0953 | 0.77±0.04 | 0.66±0.00 | 0.68±0.00 | 0.62±0.00 | 0.67±0.00 | 0.47±0.02 | 0.43±0.02 | 0.62±0.02 | 0.43±0.00 |
| 24 h *- L. fermentum* QUT 0954 | 0.81±0.03 | 0.68±0.02 | 0.65±0.02 | 0.62±0.02 | 0.65±0.00 | 0.44±0.02 | 0.44±0.02 | 0.58±0.02 | 0.34±0.02 |
| 24 h *- L. fermentum* QUT 0974 | 0.70±0.02 | 0.64±0.00 | 0.65±0.00 | 0.58±0.01 | 0.63±0.06 | 0.40±0.02 | 0.39±0.01 | 0.58±0.01 | 0.36±0.03 |
| 24 h *- L. fermentum* QUT 1057 | 0.95±0.04 | 0.82±0.00 | 0.79±0.00 | 0.68±0.00 | 0.80±0.01 | 0.59±0.02 | 0.55±0.02 | 0.73±0.02 | 0.63±0.00 |
| 24 h *- Bacillus* H57 | 0.86±0.01 | 0.78±0.10 | 0.78±0.01 | 0.75±0.06 | 0.77±0.10 | 0.64±0.02 | 0.38±0.01 | 0.59±0.02 | 0.45±0.05 |
